# The Rosetta Stone Hypothesis-Based Interaction of the Tumor Suppressor Proteins Nit1 and Fhit

**DOI:** 10.3390/cells12030353

**Published:** 2023-01-17

**Authors:** Sonnhild Mittag, Franziska Wetzel, Sebastian Y. Müller, Otmar Huber

**Affiliations:** 1 Institute of Biochemistry II, Jena University Hospital, Friedrich Schiller University Jena, Nonnenplan 2-4, 07743 Jena, Germany; 2 System Biology/Bioinformatics, Leibniz Institute for Natural Product Research and Infection Biology, Beutenbergstr. 11a, 07745 Jena, Germany

**Keywords:** Fhit, Nit1, NitFhit, Rosetta Stone hypothesis, tumor suppressor, Hsp60, Ubc9

## Abstract

In previous studies, we have identified the tumor suppressor proteins Fhit (fragile histidine triad) and Nit1 (Nitrilase1) as interaction partners of β-catenin both acting as repressors of the canonical Wnt pathway. Interestingly, in *D. melanogaster* and *C. elegans* these proteins are expressed as NitFhit fusion proteins. According to the Rosetta Stone hypothesis, if proteins are expressed as fusion proteins in one organism and as single proteins in others, the latter should interact physically and show common signaling function. Here, we tested this hypothesis and provide the first biochemical evidence for a direct association between Nit1 and Fhit. In addition, size exclusion chromatography of purified recombinant human Nit1 showed a tetrameric structure as also previously observed for the NitFhit Rosetta Stone fusion protein Nft-1 in *C. elegans*. Finally, in line with the Rosetta Stone hypothesis we identified Hsp60 and Ubc9 as other common interaction partners of Nit1 and Fhit. The interaction of Nit1 and Fhit may affect their enzymatic activities as well as interaction with other binding partners.

## 1. Introduction

**Figure 1:**
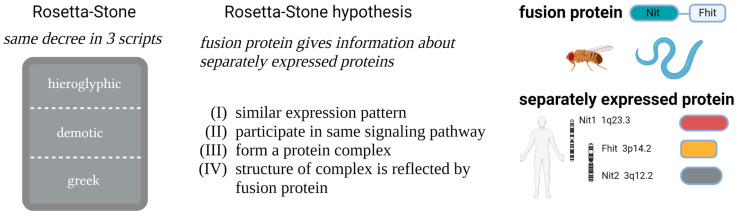
Nit1/Fhit and the Rosetta Stone hypothesis: history, postulates, and Nit/Fhit proteins (created with BioRender.com; accessed on 30th November 2022).

During evolution, chromosomal fusion or fragmentation events have generated proteins that are expressed as fusion proteins in some organisms and as separate proteins in others. Based on this observation, bioinformatic tools were developed to predict protein–protein interactions by a method of pairwise analysis of non-related proteins [1,2]. This approach was named Rosetta Stone method in reference to an ancient stone found close to the Egyptian city of Rosetta with inscriptions in Egyptian hieroglyphs, in Demotic and Greek language. The understanding of the latter languages finally helped to identify the unknown Egyptian hieroglyphs. In this context, it was postulated that the fusion proteins representing so-called Rosetta Stone proteins can provide information about the functional role of the individual proteins [3]. Based on the Rosetta Stone hypothesis, two proteins—which are expressed from separate genes—should (i) show similar expression patterns, (ii) participate in common signaling pathways or metabolic reactions, and (iii) form common protein complexes. Moreover, it is postulated that (iv) the fusion protein structurally reflects the association of the individual proteins within the common complex (Figure 1) [3].

During the identification and characterization of the human tumor suppressor protein Fhit (fragile histidine triad), it was recognized that in *D. melanogaster* and *C. elegans* Fhit is expressed as a fusion protein with a protein belonging to the nitrilase superfamily. Sequence alignment revealed human and mouse Nit1 (Nitrilase1) as closest orthologs [4]. The NitFhit fusion protein of *C. elegans* consists of four central Nit domains forming the central Nit core. The antiparallel C-terminal NS13 β-strands of each Nit domain mediates anti-parallel east–west interactions and additionally show extensive interfaces with the Fhit domains of the monomers [5]. Structurally, this orients the Fhit domains within the *C. elegans* NitFhit fusion protein in a way that they form dimers at the opposing poles of the central Nit domain tetramer, highly similar to the structure of the human Fhit dimers [6].

The human Fhit gene encoded by the FRA3B locus on chromosome 3p14.2 is frequently targeted by chromosomal aberrations and epigenetic inactivation. Impaired Fhit protein expression is detected in many of the common human epithelial neoplasias. In this context, a correlation between loss of Fhit expression and tumor progression and lymph node metastasis was reported [7]. Meanwhile, the absence of Fhit is regarded as a prognostic marker for poor outcome in different cancers, e.g., breast cancer [8]. Fhit represents a small protein of 147 amino acids with a characteristic His-X-His-X-His-XX (X: hydrophobic amino acid) motif and is thus classified as a member of the histidine-triad (HIT) protein family. Like all members of the HIT protein family, Fhit exhibits a nucleotide-binding and -hydrolyzing activity, preferentially for diadenosine-polyphosphates (ApnA, n = 3 or 4) [9]. Interestingly, the enzymatic activity of Fhit is not required for its tumor suppressive function. The mutated, enzymatic inactive FhitH96N protein binds—but does not hydrolyze—Ap_n_A. Reexpression of Fhit and FhitH96N in different Fhit-negative tumor cells as well as analysis of Fhit knock-out mice provided clear evidence for a tumor suppressive activity of both wild-type Fhit and the enzymatic-dead mutant [9,10]. The tumor suppressive function of Fhit was attributed to its pro-apoptotic activity and its role in oxidative and replicative stress or DNA damage response [9]. Moreover, the phosphorylation of Fhit at Y114 by Src-kinase reduces the hydrolase activity and stabilizes an Ap_3_A-Fhit complex, which is discussed as the active signaling form of Fhit [11,12]. Interestingly, only wild-type Fhit, but not Fhit-Y114F can induce apoptosis [13,14]. In addition, Ap_3_A-hydrolysis by Fhit can be inhibited by the interaction with Ubc9 [15,16].

Consistent with the Rosetta Stone hypothesis, Semba et al. reported an overlapping expression pattern of Fhit and Nit1 in mouse tissue. Nit1^−/−^ mice showed a phenotype comparable to Fhit^−/−^ mice. Moreover, kidney cells from Nit1^−/−^ mice showed enhanced proliferation and cyclin D1 expression. Overexpression of Nit1 similar to Fhit overexpression resulted in enhanced apoptosis. The caspase-3-dependent apoptosis induced by overexpression of Fhit was more effective in Nit1fl/fl mouse kidney cells than in Nit1^−/−^ cells and overexpression of Nit1 induced caspase-dependent apoptosis in A549 (Fhit-positive) and H1299 (Fhit-negative) human lung carcinoma cells [17].

Mammalian Nit1, Nit2, and the NitFhit fusion proteins of *D. melanogaster* and *C. elegans* are members of the 10th out of 13 branches of the nitrilase protein superfamily [18]. Mammalian Nit2 was characterized as ω-amidase, whereas Nit1 was reported to cleave several synthetic dipeptide substrates, but has no significant amidase activity [19,20]. Interestingly, in 2017 mammalian Nit1 was defined as metabolite repair enzyme that hydrolyzes deaminated glutathione [21]. Similar to Fhit, an enzymatic inactive Nit1 protein with a Cys to Ala mutation within the Glu-Lys-Cys catalytic triad revealed a tumor suppressor activity comparable to wildtype Nit1 [17]. The human nitrilases, as well as Fhit, are mainly localized in the cytoplasm, but were also detected in the nucleus and in mitochondria [17,22,23,24].

Meanwhile, there are many hints that Nit1 and Fhit are indeed physiologically linked. The deletion of both genes in mice revealed an additive tumor promotive effect, developing more spontaneous and carcinogen-induced tumors compared to single knock-out animals [25]. Previously, we identified Fhit and Nit1 as β-catenin binding-partners thereby acting as negative regulators of the canonical Wnt/β-catenin pathway [22,24]. Consistent with the mouse studies, we observed an additive repressive effect of Fhit and Nit1 on β-catenin-mediated transcriptional regulation [22]. Apart from β-catenin the transcription factors ZFYVE9 (Sara) and Smad3 were reported to interact with Nit1 [26]. However, up to now a direct interaction of Fhit and Nit1 as predicted by the Rosetta Stone hypothesis has not been shown. Here, we tested the postulated interaction of the evolutionary conserved proteins Fhit and Nit1 using biochemical analyses, including co-immunoprecipitation, in vitro pull-down assays with purified recombinant proteins, and proximity ligation assays. Additionally, we observed direct binding of Nit1 with known Fhit interaction partners Hsp60 [23] and Ubc9 [15,16], suggesting that both proteins share common interaction partners.

## 2. Materials and Methods

### 2.1. Cell Culture and Antibodies

As described previously HEK-293 (obtained from the German Collection of Microorganisms and Cell Culture GmbH) and MCF-7 (obtained for Prof. Rolf Kemler, Max Planck Institute of Immunobiology and Epigenetics) cells were grown in DMEM supplemented with 10% (v/v) fetal calf serum (PAN-Biotech, Aidenbach, Germany) and 1% (v/v) Pen/Strep solution (PAN-Biotech, Aidenbach, Germany) at 37 °C and 5% CO_2_ [27]. Monoclonal anti-FLAG M2, anti-maltose-binding protein (MBP) (clone MBP-17), and mouse monoclonal anti-tubulin (clone TUB 2.1) antibodies were purchased from Sigma (Taufkirchen, Germany). Polyclonal anti-GST antibody was a generous gift from Prof. Dr. Jürgen Wienands, University of Göttingen. Anti-Fhit (G4) was from Santa Cruz Biotechnology (Santa Cruz, Heidelberg, Germany). A polyclonal anti-Nit1 antibody was generated as reported previously [22]. Anti-myc (clone 9E10) was purified from hybridoma culture supernatant. HRP-labeled goat anti-mouse and anti-rabbit antibodies were purchased from Dianova (Hamburg, Germany).

### 2.2. Plasmids

Cytosolic Nit1 isoform 4 (NM_001185094.1) and Nit1 isoform 1 containing a mitochondrial targeting sequence (NM_005600.2) were used in this study. Human Nit1 isoform 4 and Fhit constructs were generated in previous studies [22,24]. A deletion of the 13 C-terminal amino acids of Nit1 (Nit1∆C) was obtained by PCR using oligonucleotides 5′-GCG GGA TCC TTA CAC AGG CAG GTG TCG-3′ or 5′-GCG GGA TCC CAC AGG CAG GTG TCG GCG-3′. Site-directed mutagenesis to generate catalytically inactive Nit1 variant (C167A) were performed with QuikChange site-directed mutagenesis kit (Agilent Technologies, Waldbronn, Germany) using oligonucleotides: 5′-GGC AAG ATT GGT CTA GCT GTC GCC TAT GAC ATG CGG TTC CCT GAA-3′ and 5′-GTT CAG GGA ACC GCA TGT CAT AGG CGA CAG CTA GAC CAA TCT TGC-3′. The PCR products were cloned into plasmids pCS2+, pCS2+-myc_6_, pFLAG-CMV4, pQlinkG, and pMAL-c2x. Nit1 isoform1 was cloned by standard procedure using 5′-GCG GAA TTC AGA TCT GCC ACC ATG CTG GGC TTC ATC ACC AGG-3′ and 5′-GGA TCC AGA TCT TTA AGA CAG TGG GTG ACC CAG-3′ and cloned into pMAL-c2x.

DNit and dFhit were amplified from dNitFhit (described in [22]) using oligonucleotides 5′-CGC GGA TCC GCC GCC ATG TCA ACT CTA GTT AAT ACC-3′ and 5′-C TTA ACA GCC TAC AAC CTT GCT TAA GGA TCC CGC-3′ (dNit) or 5′-GCG GGA TCC GCC GCC ATG ACC CAG GAT CGA CCA TTT G-3′ and 5′-CTG ACG GAC ATA AGC TAG GGA TCC GCG-3′ (dFhit). TEV (tobacco etch virus protease)-protease encoding plasmid pRK792 (#8830) and Hsp60 cDNA (#111662, MAC_C_CH60) were obtained from Addgene (Cambridge, MA, USA). Hsp60 was cloned into pGEX-4T1 using the following oligonucleotides 5′-GCG GGA TCC CTT CGG TTA CCC ACA GTC-3′ and 5′-GCG GGA TCC TTA GAA CAT GCC ACC TCC CAT ACC-3′. Mouse Ubc9 cDNA was subcloned from pCS2+-Ubc9-myc_6_ [28] into pGEX-4T1 using 5′-GCG GGA TCC TCG GGG ATC GCC CTC AGC-3′ and 5′-GCG GGA TCC TTA TGA GGG GGC AAA CTT CTT C-3′. Sequences of all constructs were confirmed by resequencing.

### 2.3. Co-Immunoprecipitation and Western Blot Analyses

For co-immunoprecipitation experiments 8 × 10^5^ HEK-293 cells per 6-well were transiently transfected with 2 µg of pCS2+Nit1-myc_6_, pFLAG-CMV4-Nit1 variants, pCS2+Fhit-myc_6_, pFLAG-CMV4-Fhit, or pCS2+Nit1-myc_6_ in different combinations. After 48 h cells were lysed with ice-cold lysis buffer (20 mM imidazole pH 8.0, 150 mM NaCl, 2 mM MgCl_2_, 300 mM sucrose, 0.25% (v/v) Triton X-100 and Complete protease inhibitor mix (Roche, Mannheim, Germany)) for 20 min at 4 °C, scraped and centrifuged (10 min, 20.800× *g*, 4 °C). Immunoprecipitation was performed with lysate (400 µg of total protein) and 2 µg of the appropriate antibody pre-bound to Protein A Sepharose™ (GE Healthcare, Freiburg, Germany). Subsequent Western blot analyses were performed as described previously [22,24]. For Western blot analyses, antibodies were diluted in TBST (1 µg/mL anti-FLAG M2, 1:1.000 anti-myc (9E10)).

### 2.4. Expression and Purification of Recombinant Proteins

The fusion proteins were expressed in *E. coli* strains XL1 blue or BL21DE3, grown in LB-media, and induced with 1 mM IPTG for 1 h at 37 °C or for GST-TEV-Nit1 overnight at 17 °C. Pelleted bacteria were resuspended in corresponding lysis buffer. Complete protease inhibitor mix (Roche, Mannheim, Germany) was added and cells were lysed by sonication with a Hielscher UP100H Sonotrode MS7 (Hilscher Ultrasonics GmbH, Teltow, Germany) with three times 30 pulse (90%, cycle 0.5). For affinity-purification of glutathione-S-transferase (GST)-fusion proteins on glutathione (GSH)-agarose beads (Sigma, Schnelldorf, Germany), cells were washed and lysed in PBS. Maltose-binding protein (MBP)-fusion proteins after lysis were purified in 40 mM Tris/HCl pH 8.0, 100 mM NaCl on amylose resin (New England Biolabs, Frankfurt, Germany). GST- and MBP-fusion proteins were eluted with 20 mM glutathione or 20 mM maltose both dissolved in 40 mM Tris/HCl pH 8.0, 100 mM NaCl. Purified proteins were dialyzed in 20 mM Tris/HCl pH 8.0, 50 mM NaCl or PBS. For purification of TEV protease cells were lysed in 50 mM NaH_2_PO_4_, 300 mM NaCl, 10 mM imidazole pH 8.0 and applied to Ni-NTA agarose. Columns were washed with washing buffer (50 mM NaH_2_PO_4_, 300 mM NaCl, 50 mM imidazole pH 8.0) and bound proteins were eluted with elution buffer (50 mM NaH_2_PO_4_, 300 mM NaCl, 250 mM imidazole pH 8.0). TEV protease was dialyzed in 150 mM NH_4_Cl, 1 mM EDTA, 20 mM Bicin pH 8.0 and 5% (w/v) sorbitol.

### 2.5. Pull-Down Assays

For pull-down assays similar amounts of purified GST or GST-fusion proteins were incubated with MBP or MBP-fusion proteins in pull-down buffer (150 mM NaCl, 20 mM imidazole pH 8.0, 2 mM MgCl_2_, 300 mM sucrose, 0.25% (v/v) Triton X-100) or alternatively in 20 mM Tris/HCl pH 7.5, 150 mM NaCl, 2 mM MgCl_2_, 300 mM sucrose, 0.25% (v/v) Triton X-100 for 20 min at 4 °C. The detailed procedure was described previously [29].

### 2.6. Size Exclusion Chromatography (SEC)

Untagged Nit1 and Fhit proteins were generated by expression of GST tagged fusion proteins in *E. coli* BL21DE3 using pQlinkG-based [30] constructs, binding to GSH-agarose and incubation with TEV (tobacco etch virus) protease (10 µg/mL) (expressed and purified from pRK792, obtained from Addgene, Cambridge, MA, USA) in SEC buffer (50 mM Tris/HCl pH 8.0, 100 mM NaCl, 2 mM MgCl_2_) on column over night at 4 °C to remove the GST tag. The flow-through was collected, centrifuged (10 min, 20.800× *g*, 4 °C) and subsequently analyzed on a Superdex™200-HR column (Amersham Pharmacia Biotech, Freiburg, Germany) in SEC-buffer at a flow rate of 0.3 mL/min. Molecular weight standards (Sigma, Taufkirchen, Germany) were analyzed under same conditions. Dextran blue was used to determine the column void volume. Retention volume of native marker proteins β-galactosidase (464 kDa; 9.99 mL), catalase (232 kDa; 12.02 mL), bovine serum albumin Fr. V (67 kDa; 13.73 mL), ovalbumin (47 kDa; 14.63 mL), carboanhydrase (29 kDa; 15.9 mL) and lysozym (14 kDa; 20 mL) were used for the calibration curve.

### 2.7. Proximity Ligation Assay

Proximity ligation assays (PLA) [31] were performed with 5 × 10^5^ cells of stably transfected MCF-7 shNit1 knock-down clone 12 or scrambled clone described in [22]. Cells were seeded on cover slides in six-well plates. After 48 h, PLA (Duolink^®^ Assay, Olink Bioscience distributed by Sigma, Schnelldorf, Germany) was performed with polyclonal anti-Nit1 (rabbit) and anti-β-Fhit (G4) (mouse) according to the manufacturer’s recommendations. Images were taken with an inverse fluorescence microscope (Axio Observer.Z1, Zeiss, Jena, Germany) and quantified with Fiji Open Source software [32].

### 2.8. Phylogenetic Studies

To investigate each domain of the NitFhit fusion protein individually, a search with the Blast-module of Epos 0.92svn [33] was performed using the entire fusion protein, the nitrilase domain and the Fhit domain, respectively, as a template. For the fusion protein almost all significant hits (19) were kept and manually investigated for correctness, with *D. melanogaster* NitFhit serving as representative for the *Drosophila* family (or genus) as exception. For the nitrilase and Fhit domains we selected only representative sequences across the tree of life—i.e., for bacteria, plants, fungi, and in particular Bilateria. A total of 19 fusion proteins were retrieved, as well as 18 Fhit and 46 nitrilases since they are divided into two subgroups. Sequences were aligned with the T-Coffee module of Epos for the nitrilases plus the nitrilase domain of the fusion proteins. Since the appropriate substitution model was not known for reconstructing a phylogenetic tree, we employed ProtTest 2.4 [34], which resulted in the LG matrix being the most appropriate substitution model, followed by the WAG and JTT matrix. For reconstructing the phylogenetic tree, we applied a maximum likelihood as well as a Bayesian approach to minimize a methodological bias. First, the RAxML modul of Epos was used setting the WAG matrix as substitution model with 100 replicates and for the latter the MrBayes model was applied with 1,000,000 generations. To investigate the co-evolution of Nit and Fhit, respective trees in a tanglegram-approach were compared [35]. Briefly, since the same topology can be adopted by various trees, one of the trees is rearranged to maximal resemble the second tree without changing evolutionary information of the tree.

## 3. Results

### 3.1. Co-Evolution of Nit1 and Fhit

**Figure 2:**
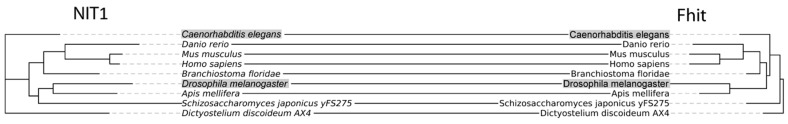
Co-evolution analysis of Nit1 and Fhit homologues genes by tanglegram-approach shown for a selected group of proteins. Fusion proteins are shaded in grey. For the complete analysis see Appendix A Figure A1A,B.

**Figure A1:**
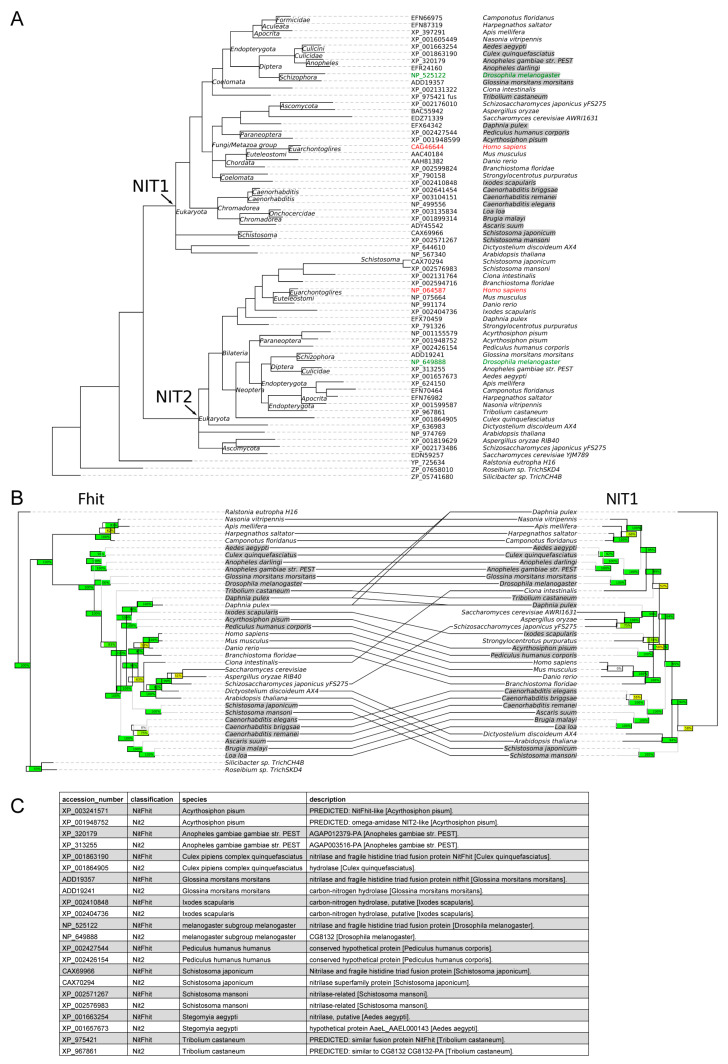
Phylogentic analysis of Nit1/2 and Fhit. For NitFhit fusion genes (shaded in grey), only the respective domain was used, i.e., the Nit domain was used to compare with non-fused nitrilases (absence of shading) and Fhit domain to compare against non-fused Fhit genes. (**A**) Pylogenetic tree of Nit1 and Nit2 orthologs across selected eukaryota. The first label column represents NCBI accession ID, second column represents the species name. (**B**) Tanglegram between Fhit genes and Nit1 genes (Nit1 subtree from A). Node colors represent bootstrap support of phylogentic tree construction. (**C**) Table of selected NitFhit fusion proteins and Nit2 homologous genes.

Initial phylogenetic analyses suggest that Nit1 and Nit2 resulted from a gene duplication (Appendix A Figure A1A). In organisms with separate Nit and Fhit genes both genes appear to co-evolve based on greatly shared phylogenetic topology (Appendix A Figure A1B). Fusion proteins can be found in some arthropoda and worms and appear to represent special events during the evolution of bilateria (Figure 2 and Appendix A Figure A1B,C). Based on sequence homologies, fusion events apparently have occurred with the Nit1, but not with the Nit2 gene. This is supported by the existence of Nit2 homologous genes in organisms with NitFhit fusion proteins (Appendix A Figure A1C). This finding supports previous studies on Nit1 and Nit2 suggesting overlapping but probably particular functions [36].

### 3.2. Human Nit1 Forms Tetramers

**Figure 3:**
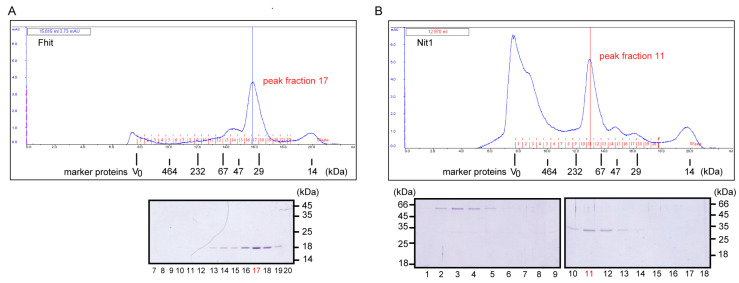
Oligomerization of Fhit (**A**) and Nit1 (**B**). Purified recombinant proteins were analyzed by size exclusion chromatography on a Superdex™200-HR column. Molecular masses of molecular weight marker proteins β-galactosidase (464 kDa; 9.99 mL), catalase (232 kDa; 12.02 mL), bovine serum albumin Fr.V (67 kDa; 13.73 mL), ovalbumin (47 kDa; 14.63 mL), carboanhydrase (29 kDa; 15.9 mL), and lysozyme (14 kDa; 20 mL) are indicated at their corresponding elution volume. Fhit (15.8 mL) and Nit1 (12.9 mL) protein in peak fractions marked in red were separated by SDS-PAGE and detected by subsequent Coomassie blue staining. The elution profiles of molecular weight standard proteins are summarized in Appendix A Figure A2.

Since the oligomeric structure of mammalian Nit1 has not been investigated up to now, we wanted to know whether human Nit1 comparable to the NitFhit fusion protein forms tetramers [5] and thus indeed differs from mammalian Nit2, which was reported to form dimers [36,37]. Therefore, Nit1 isoform 4 (~32 kDa) and human Fhit (~17 kDa) were expressed as GST-fusion proteins in *E. coli* and subsequently bound to GSH-agarose. After on-column cleavage with TEV protease the released untagged proteins were eluted and subjected to size exclusion chromatography on a Superdex™200-HR column. The peak of Fhit eluted at a volume corresponding to an apparent molecular mass of ~43 kDa. This does not ideally fit to the calculated molecular mass of ~34 kDa as expected for the dimeric structure reported in the literature [6]. However, it has to be recognized that Fhit does not reveal an ideal globular structure and moreover, the recombinant Fhit protein used in our assay includes additional amino acids remaining from the linker sequence after cleavage of GST tag by TEV protease. The peak of Nit1 eluted at a volume corresponding to a molecular mass of ~127 kDa indicating that Nit1 forms a tetramer as predicted by the Rosetta Stone hypothesis. The high molecular weight species of Nit1 that eluted around the column void volume represents uncleaved GST-Nit1 fusion protein (Figure 3).

### 3.3. Human Nit1 Forms a Complex with Fhit

**Figure 4:**
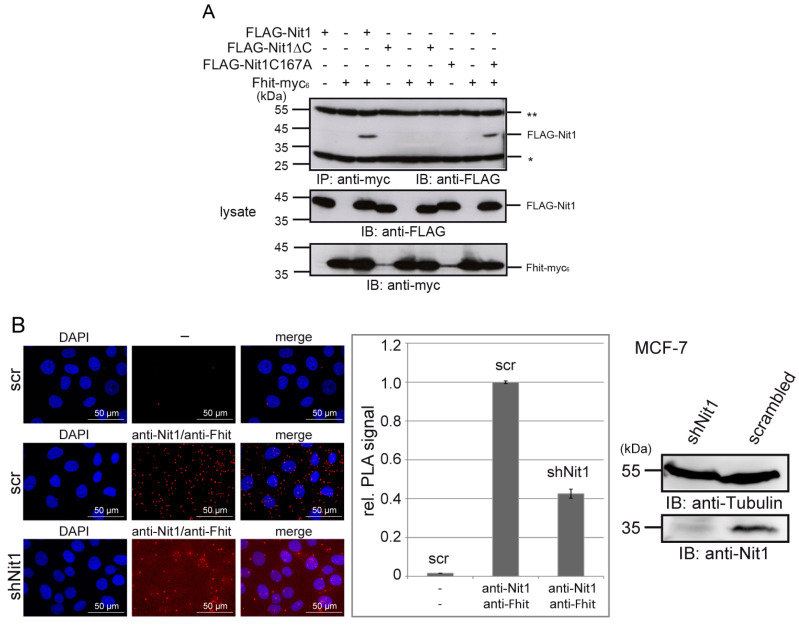
Nit1 associates with Fhit. (**A**) HEK-293 cells were transiently transfected with FLAG-Nit1 variants in combination with Fhit-myc_6_ as indicated. Fhit was precipitated with anti-myc (9E10) antibody and co-precipitated proteins were visualized by SDS-PAGE and Western blot analyses with anti-FLAG antibody. Lysate controls are shown in the lower panels. The illustrated Western blot is an example of at least three independent experiments. * light chain and ** heavy chain of precipitating antibody. (**B**) Duolink^®^ proximity ligation assays in MCF-7 cells stably transfected with scrambled or Nit1 shRNA were performed with mouse anti-Fhit and rabbit anti-Nit1 antibodies. Secondary antibody control (-) is shown in the upper part. Quantification of three independent experiments with Fiji Open Source software is presented in the bar diagram. Knock-down of Nit1 as determined by Western blotting is depicted on the right side.

**Figure A3:**
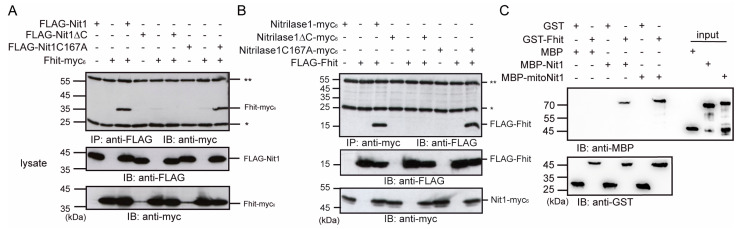
(**A**,**B**) Co-immunoprecipitation of FLAG- and myc-tagged Fhit and Nit1 variants overexpressed in HEK-293 cells as indicated. Lysate controls are shown in lower panels. The co-immunoprecipitations were performed with the same lysates as shown in Figure 4A and thus the images of the lysate controls are identical. (**C**) GST pull-down assay of Nit1 isoform 4 (MBP-Nit1) or isoform 1 (MBP-mitoNit1) with GST-Fhit. Input of MBP or MBP fusion proteins are shown at the right site. All blots are representatives of at least three independent experiments. * light chain and ** heavy chain of precipitating antibody.

Moreover, our observations that both Nit1 and Fhit act as negative regulators of Wnt/β-catenin signaling and thereby exhibit additive behavior [22,24] fulfills another central prediction of the Rosetta Stone hypothesis. However, a long-term open question is whether Nit1 can form a complex with Fhit. In this respect we first performed co-immunoprecipitation experiments with lysates of HEK-293 cells transiently transfected with FLAG-Nit1 and Fhit-myc_6_. As shown in Figure 4A (lanes 1–3) FLAG-Nit1 is specifically associated with Fhit-myc_6_ in immunoprecipitates of anti-myc antibody. Only when both constructs were co-transfected was binding of FLAG-Nit1 detectable, but not in the cells transfected with only one construct as a control. Similarly, when anti-FLAG M2 antibody was used for immunoprecipitation FLAG-Nit1/Fhit-myc_6_ complex formation was detectable (Appendix A Figure A3A). Moreover, when Nit1-myc_6_ was co-transfected with FLAG-Fhit and immunoprecipitations were performed with anti-myc antibody, association of both proteins was detectable showing that the position of the N- or C-terminal FLAG- or myc_6_-tag did not affect the result of this experiment (Appendix A Figure A3B).

In the structure of the *C. elegans* NitFhit fusion protein C-terminal β-strands in the Nit domains showed interactions with the Fhit domain dimers [5]. To test if the corresponding C-terminal sequence in Nit1 similarly contributes to the interaction of Nit1 with Fhit, a Nit1ΔC construct with a deletion of the 13 C-terminal amino acids was generated. Indeed, this construct no longer associated with Fhit in our co-immunoprecipitation experiments (Figure 4A, lanes 4–6 and Appendix A Figure A3A,B). A mutation of the active site cysteine Nit1 revealed that the catalytic activity is not required for the tumor suppressor function [17] as well as for the repressive activity on β-catenin transcriptional activity [22]. In this context, we tested if a mutated, enzymatic inactive Nit1 (Nit1C167A) protein may be impaired in binding to Fhit. However, Nit1C167A showed a similar association with Fhit as observed for wild-typ Nit1 (Figure 4A, lanes 7–9 and Appendix A Figure A3A,B). Up to now, we have not succeeded in precipitating endogenous Nit1/Fhit complexes from cell lysates as also reported in previous studies [38], suggesting that endogenous complexes are not stable enough to withstand the conditions of cell lysis, precipitation and washing steps. To circumvent this problem, we decided to use proximity ligation assay (PLA) technology, which allows localization and highly sensitive detection of endogenous proteins in close proximity [31]. As shown in Figure 4B, typical spot-like signals for an endogenous interaction of Nit1 and Fhit in MCF-7 cells were detectable. The PLA signals appear equally distributed in the cytosol with some spots also localized in the nucleus consistent with the reported nuclear localization of both proteins [17,22,24]. As a control, MCF-7 cells with shNit1 knock-down revealed significantly lower numbers of specific PLA signals.

### 3.4. Human Nit1 Directly Interacts with Fhit

**Figure 5:**
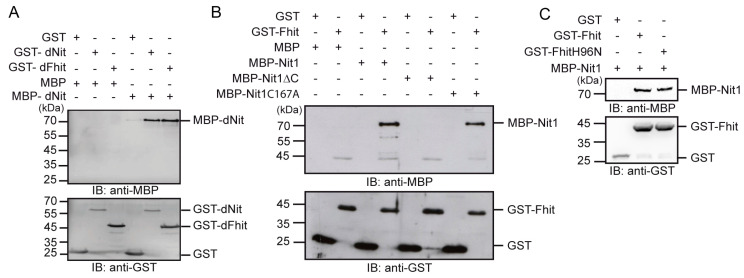
Direct interaction of recombinant Nit1 and Fhit fusion proteins. GST pull-down assays of recombinant dNit and dFhit domains of the *D. melanogaster* NitFhit fusion protein (**A**) or human Nit1 and Fhit fusion proteins as indicated (**B**). GST and MBP were used as controls. (**C**) Binding of enzymatic-dead GST-FhitH96N to wildtype MBP-Nit1. The presented blots are representatives of at least three independent experiments.

Next, we wanted to examine if the association of Nit1 and Fhit observed in the co-immunoprecipitation experiments represents a direct interaction. In a first proof of principle step we tested if the domains of the *D. melanogaster* NitFhit fusion protein can also interact when they are expressed as separate proteins. In pull-down assays with GST alone, GST-dNit or GST-dFhit in combination with MBP or MBP-dNit, a direct homophilic interaction was observed for the dNit domains as suggested in homology to the *C. elegans* NitFhit fusion protein. Moreover, GST-dNit pulled down the MBP-dFhit domain (Figure 5A).

Correspondingly, and with the aim to exclude that the observed interaction of Nit1 and Fhit in the co-immunopreciptation experiments is indirect, in vitro association assays were performed using a purified recombinant GST-Fhit fusion protein to pull down MBP-Nit1. Subsequent Western blot analyses revealed that MBP-Nit1 directly associates with GST-Fhit, whereas GST alone as a control did not bind. Consistent with the co-immunoprecipitation experiments the C-terminally deleted MBP-Nit1 protein did not interact with GST-Fhit. Again, the full-length enzymatic inactive MBP-Nit1C167A was not impaired in binding (Figure 5B). Similarly, an enzymatic inactive FhitH96N construct was not impaired in binding to Nit1 (Figure 5C). Previous studies reported that Fhit can be found in mitochondria and that overexpressed Fhit associates with mitochondrial proteins—e.g., ferredoxin reductase and Hsp60 [23]. Interestingly, one of the Nit1 transcripts encodes a protein with mitochondrial localization sequence [4,17]. Based on these observations, in a following set of experiments we wanted to know if the mitochondrial Nit1 variant (isoform 1, NM-005600.2) is also able to interact with Fhit. Using recombinant proteins we detected a direct binding of GST-Fhit with MBP-mitoNit1 in GST pull-down assays (Appendix A Figure A3C). In summary, the predictions made by the Rosetta Stone hypothesis in respect to the interactions of the separate expressed proteins Nit1 and Fhit were confirmed by our experiment.

### 3.5. Nit1 Binds to Known Fhit Interaction Partners

**Figure 6:**
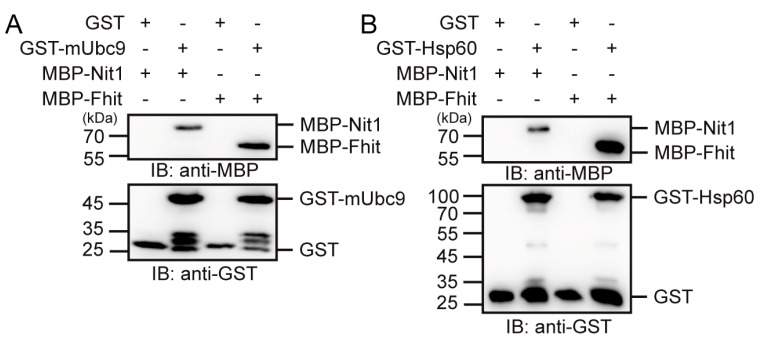
Nit1 binds to known Fhit interaction partners. MBP-Nit1 interacts with with GST-mUbc9 (**A**) or GST-Hsp60 (**B**) in pull-down assays. Protein complexes were analyzed by SDS-PAGE and subsequent Western blot analyses. The presented blots are representatives of at least three independent experiments.

The Rosetta Stone hypothesis also postulates that the separated proteins share common signaling pathways. In previous studies we identified Fhit and Nit1 as β-catenin interaction partners [22,24]. Here, we wanted to test if other known Fhit interaction partners such as Ubc9 [16] and Hsp60 [23] can also bind to Nit1. Indeed, in GST pull-down assays using purified recombinant GST-mUbc9 or GST-Hsp60 in combination with either MBP-Nit1 or MBP-Fhit, both Nit1 and Fhit associated with Ubc9 and Hsp60 (Figure 6A,B).

## 4. Discussion

Nitrilases represent a large superfamily of enzymes divided into 13 branches based on sequence analysis and anticipated substrate specificities [18]. All members are characterized by a conserved Glu-Lys-Cys catalytic triad which forms a covalent Cys-linked acylenzyme intermediate. However, only the first branch represents real nitrilases, whereas most other branches include amid-hydrolyzing or amid-condensing activities [18]. Most members of the nitrilase superfamily are of microorganismal or plant origin. The branch 10 members Nit1 and Nit2 were also identified in mammals. Human Nit1 was identified during the characterization of the tumor suppressor Fhit when it turned out that in *D. melanogaster* and *C. elegans* Fhit is expressed as a NitFhit fusion protein [4]. Subsequent database searches identified homologous human Nit1 and Nit2 proteins. Despite their homology, Nit1 and Nit2 exhibit different substrate specificities. Nit1 hydrolyzes deaminated glutathione in a metabolite repair reaction [21] and Nit2 exhibits ω-amidase activity [20,39].

Our phylogentic analyses suggest co-evolution of Nit1 and Fhit. In addition, in organisms expressing NitFhit fusion proteins, Nit2 homologs, exist. Thus, the Nit part of the fusion proteins appears to correlate with Nit1. Consistently, in our size exclusion chromatography experiments recombinant human Nit1 and Fhit form tetramers and dimers, respectively, as reported for the corresponding Nit and Fhit domains of the *C. elegans* NitFhit fusion protein [5]. Mammalian Nit2 again differs from Nit1 in forming dimers [36,37]. In addition, Pekarsky et al. observed an Ap_3_A hydrolase activity for the *D. melanogaster* NitFhit fusion protein [4]. In this context, it can be speculated that the Nit part of the NitFhit fusion protein hydrolyzes dGSH and probably does not act as an ω-amidase.

As a consequence of all these facts and according to the Rosetta Stone hypothesis, human Fhit and Nit1 proteins should form a complex, and mediate common activities in signaling or metabolic pathways as illustrated in Figure 1. In line with this, co-expression of Nit1 and Fhit was detected in animal cells [4,25], and also in *Arabidopsis thaliana*, expression of the Fhit ortholog correlates with NIT expression [40]. Moreover, an additive effect of Nit1 and Fhit double knockout on tumor formation was observed compared to single gene knockout animals [25]. Consistently, overexpression of Fhit and Nit1 augments the repressive effect of Fhit or Nit1 alone on canonical Wnt/β-catenin signaling [22].

Despite all of these facts, up to now an interaction of Nit1 and Fhit as an affirmation of one of the central postulates given by the Rosetta Stone hypothesis has not been shown. In using different experimental approaches, we here provide evidence that the long-time postulated interaction of Nit1 and Fhit indeed exists. (I) Complex formation of Nit1 and Fhit was detectable by co-immunoprecipitation from cell lysates of transiently transfected HEK-293 cell lysates. (II) Moreover, direct interaction between Nit1 and Fhit was shown in pull-down assays with purified recombinant GST- and MBP-fusion proteins. Interestingly, the C-terminal 13 amino acids of Nit1 appears to be necessary to mediate the interaction between Nit1 and Fhit as suggested from the structure of the NitFhit complex from *C. elegans* where the Nit domains assemble a central tetrameric complex with two Fhit dimers orientated into opposing directions [5]. It is interesting to speculate that the C-terminal 13 amino acids of human Nit1 may correspond to the NS13 β-strands observed in the *C. elegans* NitFhit fusion protein and thus may have an anti-parallel orientation within the Nit1 tetramers contributing to the interactions of the human Nit1 subunits. (III) Using the PLA system [31], we could observe signals for an association of endogenous Nit1 and Fhit proteins in close proximity in MCF-7 cells.

However, we were not able to co-immunoprecipitate endogenous Nit1/Fhit protein complexes. This may be due to poor binding of target specific antibodies which does not survive multiple washing steps during the co-immunoprecipitation procedure. Otherwise, the interaction of Nit1 and Fhit at endogenous levels within cells is highly dynamic and affected by specific conditions given in cells such as presence of substrates and metal ions, or may depend on posttranslational modifications including phosphorylation, ubiqitination, or sumoylation.

Regarding a potential role of substrates and enzymatic activities of Nit1 and Fhit for their interaction, it was reported that the Ap_3_A hydrolase activity of Fhit is reduced by interaction with src kinase or Ubc-9 [11,12,15,16], metal ions [41], or specific inhibitors such as Tashinone [42] or other small-molecule inhibitors [43]. Future studies have to reveal if binding of Nit1 will similarly impair the Ap_3_A hydrolase activity of Fhit or if inhibition of the Ap_3_A hydrolase activity will affect binding to Nit1. Moreover, the inhibition of the Fhit hydrolase activity is stabilizing a Fhit-Ap_n_A complex, which was predicted to be the active tumor suppressor form, and is nowadays believed to impede translation and to thereby reduce cell viability [44].

Interestingly, as cellular concentrations of diadenosine-polyphosphates (Ap_n_As) rise upon stress in plants, bacteria and also HEK-293 cells, these signaling molecules were called “alarmones” [45,46,47]. For another member of the histidine triad family, Hint1, it was reported that the binding of Ap_4_A leads to polymerization of Hint1 and disruption of the interaction with microphthalmia-associated transcription factor (MITF) [48]. This suggests that stress-dependent changes in the concentrations of Ap_3_A, Ap_4_A or even other diadenosine-polyphosphates depending on the hydrolase activities of their binding-partners can induce specific stress responses by modulating protein–protein interactions within signaling pathways. Furthermore, if Nit1 should have an effect on Fhit hydrolase activity, it vice versa has to be considered that Fhit binding to Nit1 might affect Nit1 enzymatic activity as a dGSH amidase. In our experiments, we could provide evidence that the enzymatic activity of Nit1 is not necessary for the interaction with Fhit. Enzymatic-inactive Nit1C167A still binds to Fhit in co-immunoprecipitation and pull-down asssays. In addition, the mutated FhitH96N was still able to bind Nit1 in GST pull-down assays.

The very central but still open questions that need to be solved are: in which signaling pathways are Nit1 and Fhit involved, how are these signaling pathways affected by their interaction, and how do their substrates or other interaction partners affect their function. Besides src-kinase, Ubc9 was reported to interact with Fhit thereby reducing its Ap_n_A hydrolase activity [15,16]. This interaction was questioned by Huebner et al. [49] but could be confirmed by our studies revealing a direct interaction of Fhit with Ubc9 in pull-down assays. Moreover, we also proved direct binding of Fhit to Hsp60 [50]. Interestingly, both Ubc9 and Hsp60 also directly bind to Nit1. Hsp60 is known to localize in mitochondria and to support folding and trafficking of proteins. Besides its chaperone function, Hsp60 is important in cell survival and apoptosis [51]. Moreover, Hsp60 is discussed as a biomarker for diagnosis of cancer and potential target for cancer treatment [52,53]. The physiological relevance of Nit1 interaction with Ubc9 or Hsp60 has to be analyzed. Vice versa, the interactions of Nit1 with SARA and SMAD2/3 in colorectal cancer reported by Lin et al. [26] leaves open the question as to whether Fhit can also bind to SARA to effect TGFβ-SMAD2/3 signaling.

Taken together, our results provide further evidence for the valuable information that can be deduced from the Rosetta Stone hypothesis shown here for Nit1 and Fhit. The most interesting question, however, will be: does binding of the substrates dGSH or Ap_n_A and the corresponding enzymatic activities of Nit1 and Fhit affect binding to interaction partners and thus modulate important signaling pathways such as Wnt or TGFβ signaling. In consequence, metabolic side products such as dGSH and Ap_n_As may have more important physiological or pathophysiological roles than expected.

## Figures and Tables

**Figure A2:**
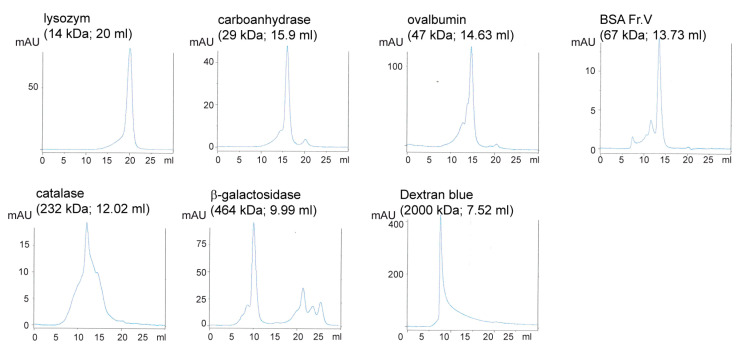
Gel filtration chromatograms of molecular weight standard proteins separated on a Superdex™200-HR column.

## Data Availability

Not applicable.
